# Functional evidence for *SCN8A* splice-donor variant c.4419+1 A > G causing loss of function

**DOI:** 10.1038/s41439-026-00356-0

**Published:** 2026-06-29

**Authors:** Takashi Shibata, Tomoyuki Akiyama, Takuma Harasaki, Sachiko Miyamoto, Hirotomo Saitsu, Toshiki Takenouchi

**Affiliations:** 1 Department of Pediatric Neurology, Okayama University Graduate School of Medicine, Dentistry and Pharmaceutical Sciences and Okayama University Hospital, Okayama, Japan; 2 Department of Biochemistry, Hamamatsu University School of Medicine, Hamamatsu, Japan

**Keywords:** Gene expression profiling, Epilepsy

## Abstract

We report a child with severe developmental and epileptic encephalopathy carrying a rare *SCN8A* splice‑site variant. Although its pathogenicity was initially unclear, a minigene assay demonstrated aberrant splicing, indicating a loss‑of‑function mechanism. Integrated clinical, genetic and functional evidence classified the variant as pathogenic, underscoring the importance of splicing assays for interpreting rare *SCN8A* variants.

Although whole-genome sequencing (WGS) has accelerated the identification of causative variants in developmental and epileptic encephalopathies (DEE), interpreting WGS results remains challenging, as many of the variants found are classified as variants of uncertain significance (VUS).

*SCN8A* gene encodes the voltage-gated sodium channel Nav1.6, a key regulator of neuronal excitability and action potential firing in the central nervous system. Since *SCN8A* was first reported as a causative gene for infantile epileptic encephalopathy in 2012 (ref.^1^), *SCN8A*-related disorders have been recognized as a heterogeneous spectrum ranging from severe DEE to neurodevelopmental delay without epilepsy^2–6^. Determining whether a variant results in gain-of-function (GOF) or loss-of-function (LOF) is clinically important, as sodium channel blockers are considered optimal first-line therapy for GOF variants, whereas they may be relatively contraindicated in LOF cases^3,6^.

In this report, we describe a patient carrying a to our knowledge previously uncharacterized *SCN8A* splice-site variant. We identified NC_000012.12(NM_001330260.2):c.4419+1 A > G, a canonical donor‑site variant that had been submitted to ClinVar as pathogenic but was classified as a VUS according to American College of Medical Genetics and Genomics (ACMG) guidelines. To clarify its functional impact, we performed a minigene splicing assay.

A 19-month-old girl was referred to Okayama University Hospital because of recurrent generalized myoclonic jerks and seizures that began with rightward eye deviation and evolved into generalized tonic–clonic seizures. She had no perinatal complications, but developmental delay had been noted since infancy. Previous evaluations at a local hospital, including blood tests, brain MRI, electroencephalography (EEG) and chromosomal G-banding (46,XX), revealed no abnormalities.

At admission, she was able to visually track objects but had poor head control and was unable to sit independently. She had no meaningful words and produced no babbling. Muscle tone in all extremities was decreased. EEG showed high-amplitude slow waves predominantly in the bilateral occipital regions, with multifocal epileptiform discharges most prominent in the bilateral posterior temporal to occipital areas (Fig. 1). Based on ictal EEG findings, the recurrent generalized jerks were considered myoclonic seizures, and the seizures beginning with ocular deviation were interpreted as focal-onset bilateral tonic–clonic seizures. Despite extensive diagnostic evaluations, no underlying disorder was identified. Valproic acid (VPA) was initiated for epilepsy, but the seizures were not controlled. At 24 months, follow-up brain MRI demonstrated progressive cerebral atrophy, raising concern for VPA-associated pseudo-atrophy. VPA was discontinued, although the atrophy did not improve thereafter.

**Fig. 1:**
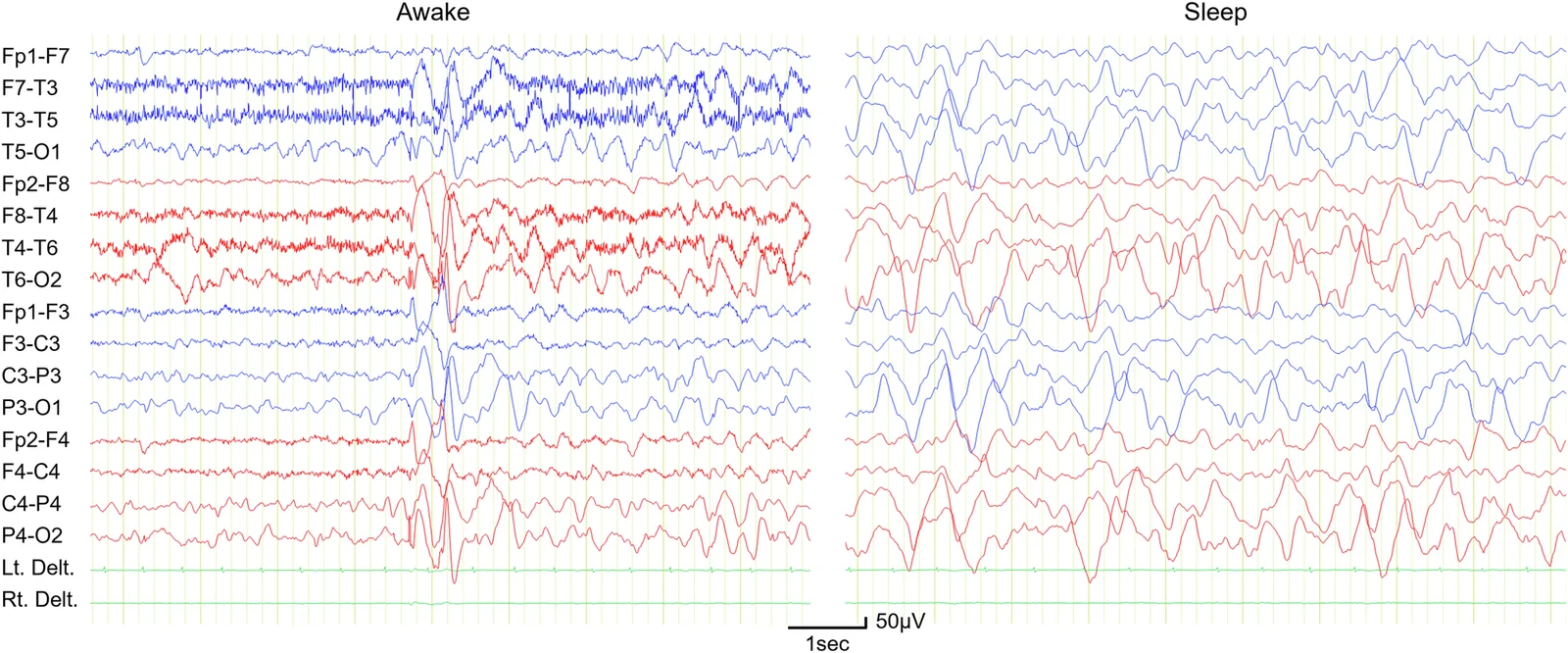
Electroencephalography findings at seizure onset. Bipolar longitudinal montage electroencephalography traces, with blue channels representing the left hemisphere and red channels representing the right hemisphere. The left panel shows the recording during wakefulness, and the right panel shows the recording during sleep. During wakefulness, posterior-dominant rhythmic activity was absent, and sporadic multifocal epileptiform discharges were observed, most prominently in the bilateral posterior temporal to occipital regions. Frequent slow‑wave activity was present throughout the recording, and upon transition to sleep, high‑amplitude slow waves became prominently increased across the entire cortex.

Her epilepsy remained refractory, and she occasionally developed status epilepticus characterized by generalized tonic–clonic seizures followed by prolonged impaired consciousness with recurrent myoclonic seizures. From around 3 years of age, myoclonic seizures were triggered by tapping stimuli to the anterior chest. However, these stimulus-induced myoclonic seizures disappeared by 4 years and 6 months.

Among antiseizure medications, clonazepam and levetiracetam were effective, whereas zonisamide, topiramate, perampanel and potassium bromide were ineffective. Lacosamide worsened her seizures and was discontinued.

To investigate the genetic basis, WGS was performed at 6 years 8 months of age, using genomic DNA extracted from the patient’s peripheral leukocytes. PCR-free libraries were sequenced using DNBSEQ-G400 (MGI TECH, China) with 150-bp paired-end reads. Variant calls were performed as previously described^7^. This analysis identified a heterozygous splice-site variant, NM_001330260.2:c.4419+1 A > G, which was confirmed by Sanger sequencing, absent in both parents and therefore considered de novo (Fig. 2A). The variant had been interpreted as pathogenic in a ClinVar submission (Variation ID: 419208). A literature search identified three reports describing the same variant, but all appeared to represent a single individual, indicating that only one case has been documented to date^4,8,9^. The NM_001330260.2:c.4419+1 A > G variant affects a rare AT–AC splice-donor site and converts the native AT dinucleotide to GT, generating a canonical GT donor site. Consistent with this change, SpliceAI did not recognize the native AT donor site but predicted the variant-created GT sequence as a novel donor site with a delta score of 0.68. Therefore, we performed a minigene splicing assay to assess its effect on splicing.

**Fig. 2:**
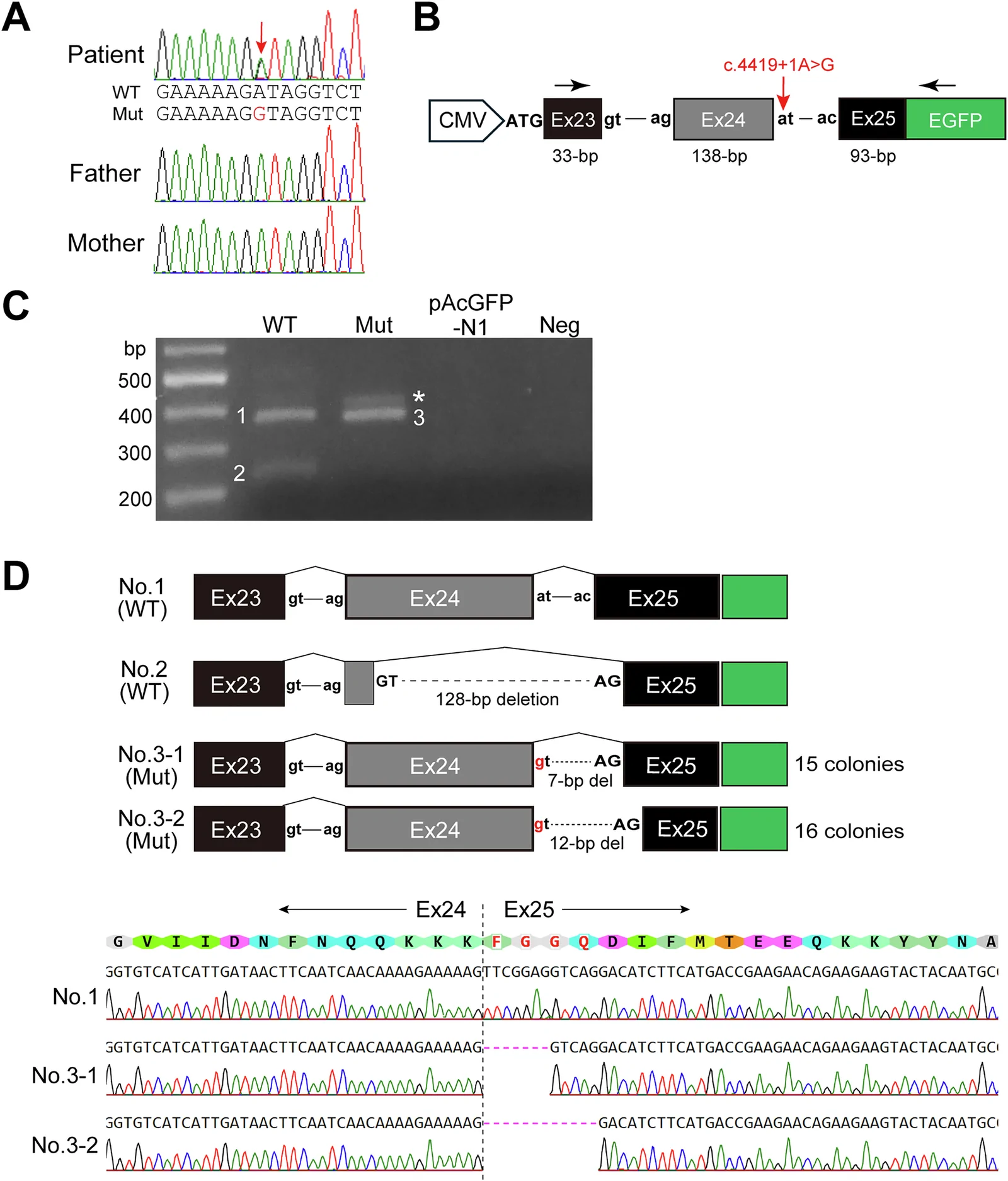
Identification and functional analysis of the *SCN8A* splice-site variant. **A** Sanger sequencing chromatograms showing the heterozygous *SCN8A* c.4419+1 A > G variant in the patient but not in either parent. The red arrow indicates the variant position. **B** Schematic representation of the *SCN8A* minigene construct containing exons 23–25 and introns 23 and 24 cloned into the pAcGFP-N1 vector. An artificial start codon was introduced upstream of exon 23, and correctly spliced transcripts are expected to generate an in-frame SCN8A–GFP fusion protein. The c.4419+1 A > G variant is indicated in red, and arrows denote RT-PCR primers. **C** RT-PCR analysis of transcripts from wild type (WT) and mutant constructs transfected into Neuro2A cells. The WT construct produced the correctly spliced transcript, band No. 1, and an additional shorter product, band No. 2. The mutant construct produced a slightly shorter product, band No. 3, and a larger, probably heteroduplex, band, indicated by an asterisk. **D** Sequence analysis of RT-PCR products. WT band No. 1 corresponded to the normal transcript, whereas WT band No. 2 contained a 128-bp deletion generated by cryptic splice-site usage. The mutant construct produced only aberrant transcripts, with either a 7-bp deletion, No. 3-1, or a 12-bp deletion, No. 3-2, in exon 25. These transcripts were identified in 15 and 16 of 31 TA-cloned colonies, respectively. del, deletion; Mut, mutant; Neg, negative control.

A DNA fragment spanning exons 23–25, including introns 23 and 24, and carrying an introduced start codon upstream of exon 23, was amplified and cloned into the pAcGFP-N1 vector (Takara Clontech, Otsu, Japan). The wild type (WT) and mutant (Mut) DNA inserts were verified by Sanger sequencing. This construct is expected to produce an in-frame SCN8A-GFP fusion protein upon correct splicing of the introns (Fig. 2B). Forty-eight hours after transfection into Neuro2A cells, total RNA was extracted and reverse-transcription PCR was performed. In the WT construct, RT-PCR detected the correctly spliced product (band No. 1 in Fig. 2C) and an additional shorter product (band No. 2). In contrast, the Mut construct produced a band slightly shorter than band No. 1 (band No. 3), together with a larger band that probably represented a heteroduplex.

To characterize these products, the corresponding WT and Mut bands were subjected to TA cloning followed by sequence analysis. In the WT construct, in addition to the normal transcript corresponding to band No. 1, we identified an abnormal product with a 128-bp deletion spanning exons 24 and 25, generated by the use of cryptic splice sites within these exons (Fig. 2D). The presence of this abnormal transcript probably reflects a limitation of the minigene splicing assay. Despite this limitation, the mutant construct produced only aberrant transcripts, with either a 7-bp deletion (No. 3-1) or a 12-bp deletion (No. 3-2) in exon 25 (Fig. 2D), and no normal transcript was detected. These two aberrant transcripts were observed at nearly equal frequencies, with 15 and 16 of 31 colonies harbouring the 7-bp and 12-bp deletions, respectively. The transcript with the 7-bp deletion is predicted to undergo nonsense-mediated mRNA decay. In contrast, the transcript with the 12-bp deletion is predicted to result in an in-frame deletion of four essential amino acids, Phe1474, Gly1475, Gly1476 and Glu1477. Notably, variants affecting this region, including p.Gly1475Arg, p.Gly1475Ala and p.Gly1476Ser, are registered as probably pathogenic in the ClinVar database.

Together with the genetic evidence, this variant was classified as pathogenic according to the ClinGen variant classification guidance for the ACMG/AMP guidelines, based on the following criteria: PVS1(RNA)_Strong, PS2_Strong and PM2_Supporting.

*SCN8A*-related disorders encompass a broad phenotypic spectrum ranging from severe DEE to neurodevelopmental delay without epilepsy. Most DEE cases are associated with GOF variants, whereas LOF variants are less common and generally show a later onset of epilepsy than GOF variants^4,6^. Our patient exhibited a clearly severe DEE phenotype, with developmental delay evident from early infancy, whereas the onset of epileptic seizures at 1 year and 7 months was relatively late compared with typical GOF‑associated *SCN8A* encephalopathy. This clinical profile is consistent with previously reported LOF cases.

The splice-donor variant identified, NM_001330260.2:c.4419+1 A > G, had been reported in only one case previously, and was classified as a VUS according to ACMG guidelines. Canonical +1 splice-site variants in *SCN8A* are expected to disrupt normal mRNA processing and lead to reduced or absent Nav1.6 function. In our case, the minigene assay demonstrated loss of normal splicing, supporting a LOF mechanism. Functional evidence addressing the impact of splice‑site variants in *SCN8A* remains limited, and our findings contribute to clarifying the pathogenicity of this rare variant.

The patient’s treatment course, in which lacosamide, a sodium channel blocker, clearly worsened seizures, further supports a LOF mechanism. The concordance between the clinical course and the predicted functional consequence of the variant, as well as the finding that the only previously reported case with the same variant was also interpreted as LOF^4^, strengthens the interpretation of this case as LOF-associated *SCN8A* DEE.

This case highlights the importance of functional evaluation for splice‑site variants detected by exome sequencing, particularly when clinical features suggest a mechanism different from the more common GOF phenotype. Although minigene assays do not fully replicate neuron‑specific splicing or channel physiology, they provide valuable evidence for variant interpretation when patient‑derived tissue is unavailable.

In summary, we reported a child with severe DEE associated with a rare *SCN8A* splice-donor variant. The clinical presentation, treatment response and minigene findings collectively support a LOF mechanism. This case expands the phenotypic and molecular spectrum of *SCN8A*-related disorders and underscores the utility of functional splicing assays in resolving the pathogenicity of rare variants.

## HGV Database

The relevant data from this Data Report are hosted at the Human Genome Variation Database at https://doi.org/10.6084/m9.figshare.hgv.3672.
